# Clinical Impact of Pathologic Residual Tumor in Locally Advanced Cervical Cancer Patients Managed by Chemoradiotherapy Followed by Radical Surgery: A Large, Multicenter, Retrospective Study

**DOI:** 10.1245/s10434-022-11583-4

**Published:** 2022-03-30

**Authors:** Alex Federico, Luigi Pedone Anchora, Valerio Gallotta, Francesco Fanfani, Francesco Cosentino, Luigi Carlo Turco, Nicolo’ Bizzarri, Francesco Legge, Elena Teodorico, Gabriella Macchia, Vincenzo Valentini, Giovanni Scambia, Gabriella Ferrandina

**Affiliations:** 1 UOC Ginecologia Oncologica, Dipartimento per la salute della Donna e del Bambino e della Salute Pubblica, Fondazione Policlinico Universitario A. Gemelli, IRCCS, Rome, Italy; 2 Istituto di Ginecologia e Ostetricia, Università Cattolica del Sacro Cuore, Rome, Italy; 3 Department of Gynecologic Oncology, Gemelli Molise, Campobasso, Italy; 4 Gynecologic Oncology Unit, Department of Obstetrics and Gynecology “F. Miulli” General Regional Hospital, Acquaviva delle Fonti, Bari, Italy; 5 Radiotherapy Unit, Gemelli Molise Hospital, Campobasso, Italy; 6 Dipartimento di Scienze Radiologiche, Radioterapiche ed Ematologiche, Fondazione Policlinico Universitario A. Gemelli, IRCCS, UOC di Radioterapia, Rome, Italy; 7 Istituto di Radiologia, Università Cattolica del Sacro Cuore, Rome, Italy

## Abstract

**Background:**

Exclusive chemoradiation (E-CT/RT) represents the standard of treatment for locally advanced cervical cancer (LACC). Chemoradiation (CT/RT) followed by radical surgery (RS) may play a role for patients with a suboptimal response to CT/RT or in low-income countries with limited access to radiotherapy. Histologic assessment of residual tumor after CT/RT and RS allows accurate definition of prognostic categories.

**Methods:**

Data on patients with FIGO stages 1B2 to 4A cervical cancer managed by CT/RT and RS from June 1996 to March 2020 were retrospectively analyzed. Pathologic response on the cervix was defined as complete (pCR), microscopic (persistent tumor foci ≤ 3 mm) (pmicroR), or macroscopic (persistent tumor foci > 3 mm) (pmacroR). Lymph node (LN) residual tumor was classified as absent or present.

**Results:**

The 701 patients in this study underwent CT/RT and RS. Of the 701 patients, 293 (41.8%) had pCR, 188 (26.8%) had pmicroR, and 220 (31.4%) had pMacroR. Residual tumor was found in the pelvic lymph nodes of 66 (9.4%) patients and the aortic lymph nodes of 29 (4.1%) patients. The 5-year DFS and OS were respectively 86.6% and 92.5% in the pCR cases, 80.3% and 89.1% in the pmicroR cases, and 56.2% and 68.8% in the pmacroR cases. Among the patients with lymph node residual tumor, the 5-year DFS and OS were respectively 16.7% and 40% in the pCR cases, 35.4% and 53.3% in the pmicroR cases, and 31.7% and 31.1% in the pmacroR cases. Cervical residual tumor,, positive pelvic LNs, and positive aortic LNs were associated with worse DFS and OS in both the uni- and multivariate analyses.

**Conclusions:**

Persistence of pathologic residual tumor on the cervix and LNs after CT/RT are reliable predictors of survival for LACC patients undergoing CT/RT and adjuvant surgery.

Cervical cancer is the fourth most common malignancy in women, with more than 500,000 new diagnoses per year and a mortality rate of about 50%, worldwide.^1^ Locally advanced cervical cancer (stages 1B2 to 4A disease) (LACC) accounts for 30–40% of new diagnoses.^2,3^ In this setting, exclusive chemoradiation (E-CT/RT) represents the standard of treatment worldwide, providing 5-year overall survival rates of 60–75%, according to stage of disease.^4,5^

In recent decades, adoption of radical surgery (RS) as an alternative to intracavitary brachytherapy after chemoradiation (CT/RT) has been proposed to improve local disease control and to reduce both radiation dose and potential toxicity.^6–9^ In the phase 2 ROMA-2 study, which adopted CT/RT with a concomitant boost followed by completion surgery, we registered a pathologic complete response rate of 50.5%, and a 3-year locoregional failure rate of only a 7%.^10^

Two prospective, randomized studies investigated the efficacy of chemoradiotherapy (CT/RT) plus RS versus E-CT/RT for FIGO stage 1B2-2 cervical cancer, but the GYNECO-002 trial was prematurely closed due to poor accrual,^11^ whereas the trial by Cetina et al.^12^ failed to demonstrate a survival advantage of RS over vaginal brachytherapy after CT/RT. Despite the lack of high-quality evidences of survival advantages for patients managed by E-CT/RT versus CR/RT followed by RS,^13–15^ the latter approach still is adopted in centers with a shortage of intracavitary brachytherapy equipment or in the clinical setting of residual tumor after CT/RT.^7,16^

Completion surgery after CT/RT provides the most relevant prognostic parameter (i.e., the pathologic assessment of residual disease in primary and lymph node sites). Indeed, absence of residual disease has been associated with better outcomes in terms of disease-free and overall survival. However, only a few studies present an acceptable sample size.^11,12,17–19^

This retrospective, multicenter study aimed to investigate the impact of pathologic residual disease on clinical outcomes in a very large population of LACC patients managed by CT/RT followed by completion surgery. Analysis of clinical and histologic parameters predicting clinical and pathologic response to CT/RT as well as clinical outcomes was performed.

## Methods

After obtaining Institutional Review Board approval (CE0019561/21), we retrospectively collected data relative to cervical cancer patients referred to the Gynecologic Oncology Unit of the Catholic University of Rome and Campobasso and the Gynecologic Oncology Unit of “F. Miulli” Hospital (Acquaviva delle Fonti) Bari, Italy. The study was performed in accordance with the criteria established by the Helsinki Declaration.

The inclusion criteria specified patients older than 18 years, biopsy-proven cervical carcinoma, and FIGO stage 1B2–4A (2009 FIGO staging classification).^20^ All the patients had signed a written informed consent agreeing to be submitted to all the procedures described, and for their data to be collected. Their pretreatment workup had included clinical examination, abdomino-pelvic magnetic resonance imaging (MRI), complete blood count and measurement of liver and renal function, cystoscopy, and proctoscopy if needed.

The patients underwent preoperative CT/RT administered as whole pelvic irradiation in combination with cisplatin-based regimens (40 mg/m^2^ of cisplatin weekly or 20-mg/m^2^ 2-h intravenous infusion on days 1 to 4 and days 26 to 30 of treatment) with or without 5-fluorouracil (1000 mg/m^2^ 24-h continuous intravenous infusion on days 1 to 4 and days 27 to 30). Slightly different schemes of platinum-based chemotherapy or radiotherapy (total dose of 39.6 to 50.4 Gy) or upper border of the radiation field (L4–L5 vs L3 vertebra) were used.^21,22^ A radiation boost to metastatic lymph nodes was always adopted.

After 5 or 6 weeks from completion to CT/RT, 18F-Fluorodeoxyglucose Positron Emission Tomography/Computed Tomography (PET/CT) and abdominopelvic MRI were performed to evaluate response to treatment according to Response Evaluation Criteria in Solid Tumors (RECIST).^23^

Surgery was performed by either a minimally invasive approach (standard straight laparoscopic instrument or robotic platform) or an open approach.^24^ Radical hysterectomy and pelvic lymphadenectomy were performed for all the patients, whereas aortic lymphadenectomy was performed in case of persistence of pelvic lymph node (LN) involvement after CT/RT at imaging, intraoperative evidence of palpable or indurated or fixed pelvic and/or aortic LNs, and intraoperatively assessed involved pelvic LNs at frozen section analysis.

### Evaluation of Pathologic Response

Residual disease was evaluated based on examination of T and N. At histopathologic evaluation, the cervix was sectioned clockwise in at least 12 blocks and entirely embedded in paraffin. From each block, 3- to 4-μm-thick slides were cut at different levels and stained with hematoxylin and eosin. Histologic evaluation was performed by dedicated pathologists experienced in gynecologic oncology. Pathologic response was defined as complete (absence of any residual tumor after treatment at any site level) (pCR), microscopic (persistent tumor foci ≤ 3 mm maximum dimension) (pmicroR), macroscopic (persistent tumor foci > 3 mm maximum dimension) (pmacroR) according to the final pathology.^25^ Evaluation of pelvic/aortic LN status was described as presence versus absence of disease.

### Adjuvant Treatment

Patients achieving pCR or pmicroR started surveillance routinary procedures, whereas patients achieving macroscopic partial response (pmacroR) or involvement of pelvic and/or aortic LNs were triaged to adjuvant chemotherapy.

### Statistical Analysis

Descriptive statistics were used to summarize clinicopathologic features of the study population. Quantitative variables were described using the following measures: minimum, maximum, range, mean, and standard deviation. Qualitative variables were summarized with absolute and percentage frequency. Normality of continuous variables was checked using the Kolmogorov–Smirnov test. The chi-square test or Fisher’s exact test for proportion was used to analyze the distribution of clinical and pathologic variables among subgroups.

The primary end point was the percentage of pathologic complete response (pCR) to CT/RT on tumor tissue specimens after surgery. The secondary end points were disease-free survival and overall survival according to clinical response after CT/RT and pathologic response on tissue specimens.

Disease-free survival (DFS) was calculated from the date of surgery to the date of relapse or the date of the last follow-up visit. Overall survival (OS) was calculated from the date of diagnosis to the date of death or the date of the last follow-up visit. Survival curves were presented as Kaplan and Meier plots.^26^ Cox proportional hazard^27^ models were used to estimate the hazard ratios (HRs) and 95% confidence intervals (95% CIs) for DFS and OS. A logistic regression model was used to analyze relationships, expressed by odds ratio (OR) with 95% confidence interval, between clinical/pathologic features and dichotomous dependent variables.^28^

The Statistical Package for Social Sciences software, version 25.0 (IBM Corporation, Chicago, IL, USA) and Stata software version 13.0 (StataCorp) were used for statistical analysis. All *p* values were two-sided, and a *p* value lower than 0.05 was considered statistically significant.

## Results

**Fig. 1:**
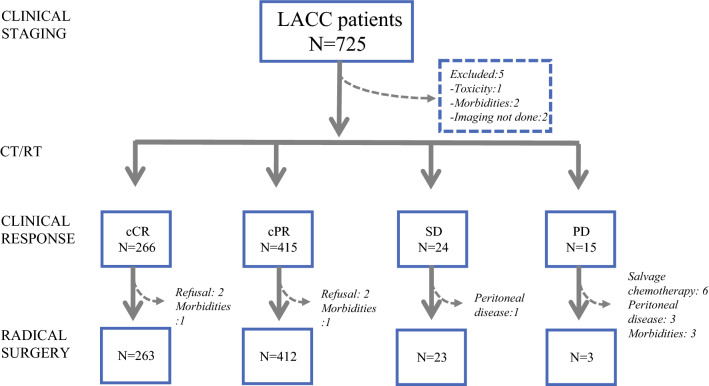
Flowchart of patients

From June 1996 to March 2020, 725 consecutive LACC patients were triaged to CT/RT (Fig. 1). Five patients were excluded from the analysis of response to CT/RT because of incomplete treatment due to morbidities (*n* = 2), severe toxicity (*n* = 1), and unavailability of imaging (*n* = 2). Consequently, 720 patients completed CT/RT. Clinical complete response (cCR) was observed in 266 (36.9%) patients and clinical partial response (cPR) in 415 (57.6%) patients, whereas 24 (3.3%) patients had stable disease (SD), and 15 (2.1%) patients had progression of disease (PD).

Table 1 summarizes the clinicopathologic features of the 720 patients. The median age was 51.5 years (range, 20–83 years). In terms of histology, 634 (88.1%) patients had squamous cell carcinoma, whereas 505 (70.1%) patients had FIGO stage 2B disease. At staging workup, lymph node status was negative in 398 (55.3%) patients, whereas 298 (41.4%) patients had only positive pelvic LNs, and only 24 (3.3%) patients had metastatic aortic plus pelvic LN involvement.

**Table 1:** Clinical and pathologic features

Variable	All (*n* = 720) *n* (%)
Median age: years (range)	51.5 (20–83)
Median BMI: kg/m^2^ (range)	23.9 (16.4–45)
FIGO stage	
1B2	60 (8.3)
2A	39 (5.4)
2B	505 (70.1)
3A	26 (3.6)
3B	79 (11)
4A	11 (1.5)
Tumor size (mm)	
< 40	125 (17.4)
> 40	595 (82.6)
Histotype	
Squamous	634 (88.1)
Adenocarcinoma	71 (9.9)
Other	15 (2.1)
Lymph node status at imaging	
Negative	398 (55.3)
Positive pelvic only	298 (41.4)
Positive aortic ± pelvic	24 (3.3)
Clinical response to CT/RT	
Complete	266 (36.9)
Partial	415 (57.6)
Stable disease	24 (3.3)
Disease progression	15 (2.1)

BMI, body mass index; CT/RT, chemoradiation

After CT/RT, radical surgery (RS) was performed for 701 patients, with no RS for the remaining 19 patients due to patient refusal (*n* = 4), morbidities (*n* = 5), intraoperative documentation of peritoneal disease (*n* = 4), or unresectable disease progression (*n* = 6).

Most of the patients underwent type 3 or 4 radical hysterectomy (73.4%). Pelvic lymphadenectomy was performed for all the patients, whereas 125 (29.7%) patients also underwent aortic lymphadenectomy. The median interval from the end of CT/RT to imaging was 4 weeks (range, 3–7 weeks), whereas the median interval from the end of CT/RT to surgery was 6 weeks (range, 4–8 weeks) (data not shown).

Table 2 shows the uni- and multivariate logistic analyses of clinical and pathologic parameters for prediction of cCR to CT/RT. Squamous histotype, stage 1B2–2B, tumor smaller than 4 cm, and negative LN status at imaging assessment kept their independent, favorable impact for prediction of cCR in the multivariate analysis.

**Table 2:** Clinical and pathologic parameters for prediction of clinical complete response

	*N*	Univariate analysis			Multivariate analysis		
		β	OR (95% CI)	*p* value	β	OR (95% CI)	*p* value
Histotype							
Squamous	634		1			1	
Other	86	0.965	2.625 (1.508–4.569)	**0.001**	1.035	2.815 (1.596–4.966)	**<** **0.001**
FIGO stage							
1B2–2B	604		1			1	
3A–4A	116	0.769	2.158 (1.362–3.421)	**0.001**	0.769	2.157 (1.357–3.455)	**0.001**
Tumor size (cm)							
< 4	125		1			1	
> 4	595	0.674	1.962 (1.329–2.896)	**0.001**	0.48	1.615 (1.076–2.425)	**0.021**
Lymph node status at imaging							
Negative	398		1			1	
Positive	322	0.538	1.712 (1.255–2.334)	**0.001**	0.467	1.596 (1.155–2.204)	**0.005**

Bold values indicate statistical significance at the *p* < 0.05 level

We analyzed the distribution of the pathologic residual disease in the primary tumor site as well as the LN stations in the 701 patients (Table 3) and found that 293 (41.8%) patients had a pathologic complete response (pCR), whereas 188 (26.8%) patients showed ≤ 3 mm residual tumor (pmicroR), and 220 (31.4%) had > 3 mm residual tumor (pmacroR). In the 293 patients with absence of cervical residual tumor, we registered only six cases (2%) with metastatic LNs (3 with only positive pelvic LNs and 3 with aortic ± pelvic LNs). Among the patients with cervical microscopic (≤ 3 mm) residual tumor, we found 13 patients with positive pelvic LNs (6.9%) and 5 patients with positive aortic plus pelvic LNs (2.7%). On the other hand, the group with macroscopic (> 3 mm) residual disease in the cervix comprised 71 patients (32.2%), including 50 patients with positive pelvic LNS and 21 patients with metastatic disease (*p* = 0.001).

**Table 3:** Histopathologic details

	All N=701	Negative LNs (*n* = 606)	Positive pelvic LNs (*n* = 66)	Positive aortic ± pelvic LNs (*n* = 29)	*p* value
Cervical residual tumor	*n* (%)^a^	*n* (%)^b^	*n* (%)^b^	*n* (%)^b^	**<** **0.001**
Absent	293 (41.8)	287 (98.0)	3 (1.0)	3 (1.0)	
< 3 mm	188 (26.8)	170 (90.4)	13 (6.9)	5 (2.7)	
> 3 mm	220 (31.4)	149 (67.7)	50 (22.7)	21 (9.5)	

^a^Percentage calculated on 701 patients

^b^Percentage calculated on the cervical residual tumor

Bold values indicate statistical significance at the *p* < 0.05 level

The patients with cervical macroscopic residual and/or persistence of metastatic pelvic and/or aortic lymph nodes after CT/RT and surgery were managed with four cycles of carboplatin (AUC, 5) and paclitaxel (175 mg/m^2^) q21 (every 21 days). For the patients older than 70 years and those with severe comorbidities, chemotherapy was modified by weekly carboplatin (AUC, 2) and paclitaxel (60 mg/m^2^) d1, d8, d15, q28 (given on days 1, 8 and 15, every 28 days).

Of 144 patients suitable for adjuvant chemotherapy, 127 were managed by carboplatin/paclitaxel q21, whereas 11 patients underwent carboplatin/paclitaxel weekly, and 6 patients refused chemotherapy. Moreover, seven patients showing positive vaginal margins after surgery were managed by intracavitary brachytherapy.

### Clinical Outcomes

As of May 2021, the median follow-up period was 56 months (range, 4–248 months), during which 183 recurrences and 126 deaths were registered. In the whole series, the 5-year DFS was 71.4%, and the OS was 79.6% (data not shown).

**Fig. 2:**
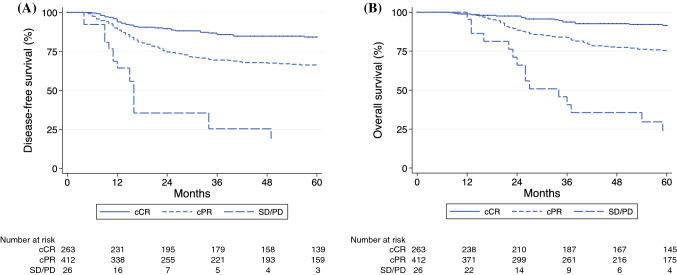
Cumulative curves for (**A**) disease-free survival (DFS) and (**B**) overall survival (OS) according to clinical response to chemoradiation (CT/RT). cCR (*solid line*), cPR (*dash line*), SD/PD (*long-dash line*)

Figure 2 shows the Kaplan-Meier curves for DFS and OS according to clinical response to CT/RT. The patients with cCR experienced a 5-year DFS of 84.2%, whereas the patients with cPR experienced a 5-year DFS of 66.5%. For the patients with SD/PD, the 5-year DFS was 19.0% (Fig. 2A). After CT/RT, the patients with cCR showed a 5-year OS of 91.5%, whereas patients with cPR exhibited a 5-year OS of 75.4%. The patients with SD/PD had a 5-year OS of 23.7% (Fig. 2B).

**Fig. 3:**
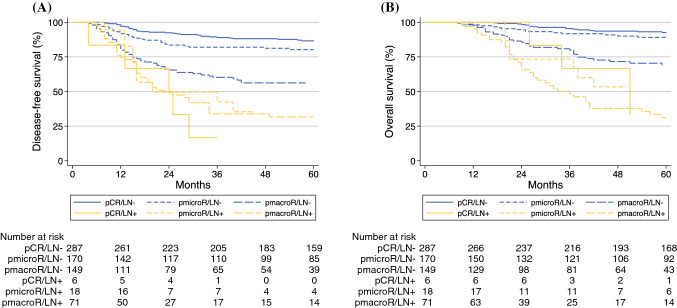
Cumulative curves for (**A**) disease-free survival (DFS) and (**B**) overall survival (OS) according to pathologic response to chemoradiation (CT/RT). Negative lymph nodes (LNs): black lines (pCR [*solid line*], pmicroR [*dash line*], pmacroR [*long-dash line*]). Positive LNs: gray lines (pCR [*solid line*], pmicroR [*dash line*], pmacroCR [*long-dash line*])

Figure 3A shows estimated DFS survival curves according to pathologic response. Among the patients with negative LNs, the 5-year DFS was 86.6% for the patients with absence of cervical disease, 80.3% for the patients with presence of ≤ 3-mm disease, and 56.2% for the patients with > 3-mm disease. In the same panel, among the patients with persistence of LN disease, the 5-year DFS was 16.7% for the patients with absence of cervical disease, 35.4% for the patients with ≤ 3-mm disease, and 31.7% for the patients with > 3-mm disease.

Figure 3B shows OS outcome according to pathologic status. Among the patients with negative LNs, the 5-year OS rate was 92.5% for the patients without cervical disease, 89.1% for the patients with ≤ 3-mm disease, and 68.8% for the patients with > 3 mm in the cervix. Among the patients with metastatic LNs, the 5-year OS was 40% for the patients without residual cervical disease, 53.3% for the patients with ≤ 3-mm disease, and 31.1% for the patients with > 3-mm disease.

Table 4 shows the uni- and multivariate analyses of age, histotype, pathologic residual tumor in the cervix, and lymph node status as prognostic parameters for DFS and OS. In the univariate analysis, absence of cervical residual tumor and negative LNs were statistically significant for better DFS and OS. The multivariate analysis confirmed that pathologic response and pathologic LN status at the time of surgery maintained their independent impact on DFS and OS.

**Table 4:** Clinical and pathologic features as prognostic factors for disease-free survival and overall survival (*n* = 701)

		Disease-free survival						Overall survival					
		Univariable			Multivariable			Univariable			Multivariable		
		$\beta$	HR (95% CI)	*p* Value	$\beta$	HR (95% CI)	*p* Value	$\beta$	HR (95% CI)	*p* Value	$\beta$	HR (95% CI)	*p* Value
Age^a^	–	− 0.085	0.919 (0.814–1.037)	0.171	− 0.098	0.907 (0.801–1.027)	0.122	− 0.063	0.939 (0.811–1.087)	0.397	− 0.097	0.908 (0.781–1.055)	0.208
Histotype													
Squamous	619		Reference			Reference			Reference			Reference	
Other	82	0.310	1.364 (0.906–2.052)	0.137	− 0.334	0.709 (0.463–1.087)	0.115	0.348	1.417 (0.870–2.308)	0.162	− 0.513	0.599 (0.357–1.003)	0.051
Pathologic residual tumor													
Absent	293		Reference			Reference			Reference			Reference	
< 3 mm	188	0.597	1.816 (1.172–2.816)	**0.008**	0.534	1.706 (1.098–2.649)	**0.018**	0.717	2.049 (1.167–3.598)	**0.012**	0.611	1.841 (1.045–3.245)	**0.035**
> 3 mm	220	1.595	4.927 (3.404–7.132)	**<** **0.001**	1.373	3.948 (2.634–5.92)	**<** **0.001**	1.925	6.858 (4.262–11.035)	**0.000**	1.613	5.016 (2.996–8.399)	**<** **0.001**
Lymph node status													
Negative	606		Reference			Reference			Reference			Reference	
Positive pelvic only	66	1.173	3.233 (2.208–4.733)	**<** **0.001**	0.580	1.786 (1.188–2.686)	**0.005**	1.471	4.355 (2.8–6.773)	**<** **0.001**	0.860	2.364 (1.478–3.78)	**<** **0.001**
Positive aortic ± pelvic	29	2.052	7.787 (5.024–12.071)	**<** **0.001**	1.616	5.035 (3.176–7.981)	**<** **0.001**	2.617	13.698 (8.494–22.098)	**<** **0.001**	2.181	8.856 (5.331–14.712)	**<** **0.001**

^a^Analysis performed for each 10-year increase in age

Bold values indicate statistical significance at the *p* < 0.05 level

## Discussion

This study analyzed the role of clinical and pathologic response to CT/RT in a large retrospective series of LACC patients who underwent CT/RT followed by radical surgery. Clinical response at imaging plays an important role in evaluation of response/persistence of cervical and/or lymph node disease after CT/RT. In this study, the patients with a clinical complete response after CT/RT experienced a 5-year DFS of 84.2%, whereas the patients with a clinical partial response had a 5-year DFS of 66.5%, and the patients with stable/progression of disease had a 5-year DFS of 19.0%. Moreover, the data relative to clinical response may help in the selection of patients deemed to be triaged to radical surgery, and could be helpful in terms of tailoring radical surgery.^24,29^

However, the clinical response to CT/RT did not show sufficient reliability, likely due to difficulty correlated with tumor necrotic tissues, post-treatment artifacts, and other radiation-induced modifications of cervical and LN tissues.^30–32^ For detection of metastatic LNs from cervical cancer, the MRI accuracy rate is 76–100%, and the sensitivity rate is 36–71%.^31,32^ Data regarding the diagnostic performances of PET/CT after CT/RT showed that almost half of the cases with negative findings at imaging exhibited the presence of residual tumor at final pathology, and that the negative predictive value on the LN status was 75.3%.^33^

In this context, completion surgery after CT/RT allows the provision of certain pathologic findings regarding the response to treatment. We noted that the pathologic response after CT/RT has a significant impact on the survival outcomes for LACC patients managed by CT/RT followed by radical surgery. In this large retrospective study, 287 patients achieved a pathologic complete response rate for the cervix and lymph nodes of about 40%, a figure consistent with previous experiences.^7–12^ These data resulted in very high 5-year DFS (86.6%), and OS (92.5%) rates. Moreover, we also registered a 5-year DFS rate of 80.3% and a 5-yearr OS rate of 89.1% for the patients with only cervical microscopic disease, thus leading to an approximate 65% rate of patients who could have experienced long-term outcomes.

Conversely, for the patients with only macroscopic cervical residual disease, the 5-year DFS was 56.2%, and the 5-year OS was 68.8%. This group could be considered as having an intermediate prognostic setting compared with the worst one (i.e., the patients with residual LN disease).

In the multivariate analysis of suggested parameters, the pathologic response on the cervix and the pathologic status of LNs maintained their independent impact on DFS and OS. However, we must acknowledge that persistence of residual disease in aortic plus pelvic LNs leads to a more unfavorable prognosis with respect to persistence of cervical disease.

Our analysis was characterized by some limitations. Because of the retrospective design, some clinical and pathologic data were lacking, such as details on the radiation therapy and, in most cases, the extension of radiation fields, as well as the size of LN residual tumors. Furthermore, the data were collected during a long-term interval, and a certain degree of inter-observer variability in residual tumor measurement could be suggested, thus limiting the reproducibility of the reported data. Conversely, with decades of experience in completion surgery after CT/RT for LACC patients, the strengths of our analysis were the large study population, the long follow-up period, and the systematic performance of pelvic lymphadenectomy, which comprehensively allowed reporting on the histologic evaluation of LN status. Furthermore, the patients were managed by relatively homogeneous chemoradiotherapy schedules and adjuvant treatments, thus minimizing the effect of potential confounders.

The results of the current study encourage clinicians to focus their efforts on patients with a non-complete response to CT/RT due to their poor survival outcomes. The INTERLACE phase 3 randomized study (NCT:01566240) is investigating the presence of neoadjuvant chemotherapy before exclusive CT/RT versus placebo. Moreover, the OUTBACK study (NCT: 01414608) trial has completed the enrollment of LACC patients managed by exclusive CT/RT followed by adjuvant chemotherapy versus placebo and at the ASCO meeting on June 2021 showed that adjuvant chemotherapy given after chemoradiation does not improve clinical outcomes.^34^

In conclusion, analysis of pathologic response to CT/RT represents one of the most relevant prognostic factors for LACC patients managed by CT/RT followed by radical surgery. It allows a reliable stratification of risk of recurrence/death from disease that could be used to select patients suitable for further therapies.
